# Optimal Look-Back Period to Identify True Incident Cases of Diabetes in Medical Insurance Data in the Chinese Population: Retrospective Analysis Study

**DOI:** 10.2196/46708

**Published:** 2023-11-06

**Authors:** Wenyi Yang, Baohua Wang, Shaobo Ma, Jingxin Wang, Limei Ai, Zhengyu Li, Xia Wan

**Affiliations:** 1 Institute of Basic Medical Sciences Chinese Academy of Medical Science Beijing China; 2 School of Basic Medicine Peking Union Medical College Beijing China; 3 Chinese Center for Disease Control and Prevention National Institute for Prevention and Control of Chronic Noncommunicable Diseases Beijing China; 4 Weifang Medical Insurance Center Weifang China; 5 Department of Clinical Medicine Qingdao University Medical College Qingdao China

**Keywords:** diabetes, incident cases, administrative data, look-back period, retrograde survival function

## Abstract

**Background:**

Accurate estimation of incidence and prevalence is vital for preventing and controlling diabetes. Administrative data (including insurance data) could be a good source to estimate the incidence of diabetes. However, how to determine the look-back period (LP) to remove cases with preceding records remains a problem for administrative data. A short LP will cause overestimation of incidence, whereas a long LP will limit the usefulness of a database. Therefore, it is necessary to determine the optimal LP length for identifying incident cases in administrative data.

**Objective:**

This study aims to offer different methods to identify the optimal LP for diabetes by using medical insurance data from the Chinese population with reference to other diseases in the administrative data.

**Methods:**

Data from the insurance database of the city of Weifang, China from between January 2016 and December 2020 were used. To identify the incident cases in 2020, we removed prevalent patients with preceding records of diabetes between 2016 and 2019 (ie, a 4-year LP). Using this 4-year LP as a reference, consistency examination indexes (CEIs), including positive predictive values, the κ coefficient, and overestimation rate, were calculated to determine the level of agreement between different LPs and an LP of 4 years (the longest LP). Moreover, we constructed a retrograde survival function, in which survival (ie, incident cases) means not having a preceding record at the given time and the survival time is the difference between the date of the last record in 2020 and the most recent previous record in the LP. Based on the survival outcome and survival time, we established the survival function and survival hazard function. When the survival probability, S(t), remains stable, and survival hazard converges to zero, we obtain the optimal LP. Combined with the results of these two methods, we determined the optimal LP for Chinese diabetes patients.

**Results:**

The κ agreement was excellent (0.950), with a high positive predictive value (92.2%) and a low overestimation rate (8.4%) after a 2-year LP. As for the retrograde survival function, S(t) dropped rapidly during the first 1-year LP (from 1.00 to 0.11). At a 417-day LP, the hazard function reached approximately zero (h_t_=0.000459), S(t) remained at 0.10, and at 480 days, the frequency of S(t) did not increase. Combining the two methods, we found that the optimal LP is 2 years for Chinese diabetes patients.

**Conclusions:**

The retrograde survival method and CEIs both showed effectiveness. A 2-year LP should be considered when identifying incident cases of diabetes using insurance data in the Chinese population.

## Introduction

Diabetes is a severe, long-term disease that significantly impacts the lives of individuals, families, and societies worldwide [1]. In the past 3 decades, the prevalence of type 2 diabetes has risen dramatically. About 422 million people worldwide have diabetes, and 1.5 million deaths are directly attributable to diabetes each year, as reported by World Health Organization in 2022. In 2013, China had the largest number of patients with diabetes and the second-highest spending on diabetes and its complications worldwide [2,3]. Therefore, to achieve global agreement and halt the worldwide rise in diabetes by 2025, it is crucial to prevent and control diabetes in China.

An accurate estimate of incidence and prevalence plays a crucial role in properly preventing and controlling diabetes. Although large-scale and representative surveys could be used for precise estimation of incidence [4-6], they are expensive, laborious, and time-consuming. Currently, only a few areas have established incident case surveillance systems for chronic diseases in China, and only for some chronic diseases (such as diabetes and hypertension), with short monitoring periods and few sample surveillance points, which limits their usefulness. An alternative method is to use administrative data, such as medical insurance data or disease registration data. Advantages of using these administrative data include easy access, low cost, and large sample size (covering the majority of residents), as well as the ability to follow up with participants longitudinally for many years; this has been confirmed by many researchers in other countries [7-11]. Since medical insurance reforms in 2012 [12], China has vigorously promoted medical policies and expanded coverage of the population [13]. Effective data have gradually accumulated during the past few years, making it possible to conduct research in this population.

However, multiple records of the same patient make it a general challenge to identify true incident cases through medical insurance data, as it is difficult to define the starting point of certain diseases for a patient. Before the index year, a look-back (LP) period is used to deal with this problem. Patients with a diabetes diagnosis in the index year and without a diabetes diagnosis in the LP would be identified as incident cases. Until now, there has been no consensus on the length of the LP. An insufficient LP might cause overestimation of incidence [14], whereas an overlong LP would limit the number of reporting years and the usefulness of the database. Researchers have found that different diseases have different LPs due to specific trajectories [11]. For diabetes, Brameld et al [15] identified an LP of 13 years using data from the Western Australian Data Linkage System, while Asghari et al identified an LP of 5 years using data from the Régie de l’assurance maladie du Québec [7]. We believe that different patterns of medical contact cycles among countries may cause these different findings on LPs. However, there is no study on the optimal LP in the Chinese population for diabetes.

In this study, we will use 2 methods to identify the optimal LP for diabetes in the Chinese population, using data from an insurance database for 2016 to 2020 obtained from the Medical Security Bureau (MSB) of the city of Weifang, China, as an example. Weifang, a prefecture-level city in Shandong province with 9.4 million long-term residents, has a systematic method for ensuring medical service, and the coverage rate for medical insurance reached 96.15% in 2021. The aim of this study is to identify the optimal LP for diabetes for the Chinese population and additionally to compare different results by using different methods to obtain a reference for other diseases in the administrative data.

## Methods

### Ethical Considerations

The proposal for this study was reviewed by Chinese Academy of Medical Science & School of Basic Medicine (033-2018). The insurance claims data were obtained from the MSB of Weifang. In order to protect the privacy of patients, all personally identifiable information in the database is obfuscated.

### Data Sources

The database used in this study included all in- or outpatient claim records of patients in Weifang for 2016 to 2020 provided by the local MSB. The social health insurance system covered approximately all residents in Weifang (close to 9 million individuals) during the period from 2016 to 2020. Each claim record contains (1) basic information about the patient, including personal identification number, gender, date of birth, and type of medical insurance; (2) medical information, including hospital name and code, admission and discharge time, and diagnosis of disease; and (3) details of medical service expenses, including total expenses, out-of-pocket expenses, and reimbursement expenses.

### Data Processing

**Figure 1:**
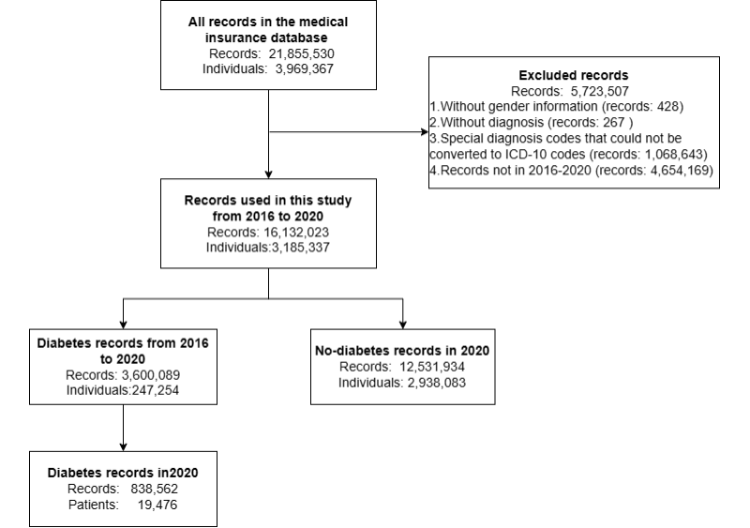
Flow chart showing included and excluded records and the process used for data cleaning of the insurance claim data (January 1, 2016, to December 31, 2020). ICD-10: International Classification of Disease, 10th Revision.

Firstly, due to the changes in the Weifang disease coding system and the special disease codes in outpatient chronic-disease records, the initial disease codes in the database were partly based on the International Classification of Diseases, 10th Revision (ICD-10). Therefore, the diagnoses were harmonized with the ICD-10 according to the code-disease mapping table provided by the MSB of Weifang. Later, records with critical missing information, such as gender (418/21,855,530, <0.001%) or disease diagnosis (1,068,910/21,855,530, 4.89%), were deleted. The data processing is shown in Figure 1.

### Definition of Incident Diabetes for the Application of Different LPs

First, we defined diabetes (ICD-10 codes E10-E14) cases as those with at least 1 inpatient claim record or 3 outpatient claim records with a diagnosis of diabetes, considering that inpatient records are more reliable than outpatient records [16]. In this database, we found that all patients defined as having diabetes had more than 3 outpatient records, reflecting the regularity of medical contact.

**Figure 2:**
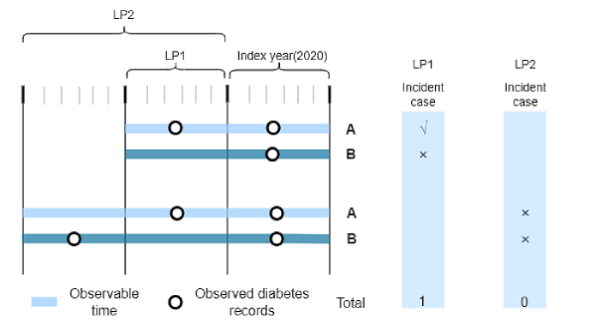
Definition of incident cases under 2 scenarios. Scenario 1 (LP1): patient B will be identified as an incident case but patient A will not; scenario 2 (LP2): both patient A and patient B are not identified as incident diabetes cases. LP: look-back period.

Second, we defined incident diabetes cases. The procedure to identify incident cases was to exclude cases with any in- or outpatient records of diabetes in the given LP, before the index year. Different LPs may classify patients as having different statuses. Two scenarios for assumed different LPs are shown in Figure 2. Scenario 1 (LP1) shows that patient B will be identified as an incident case but patient A will not. Scenario 2 (LP2) shows that with the extension of the LP, more records of patient B might be caught, but patient A and patient B will both be identified as prevalent cases. In order to use as long an LP as possible in this study, we used 2020 as the index year and a 4-year LP (2016-2019) as the longest LP.

### Statistical Analyses

Two methods were developed to identify the optimal LP. First, consistency examination indexes (CEIs) were used to determine the level of agreement between different LPs from a quarter of a year to 4 years. Second, a retrograde survival function method was used. Finally, the optimal LP was selected after considering the results from the above approaches.

The CEIs include the κ value, positive predictive values (PPVs), and overestimation value. The relative formulas have been reported in other papers [4,15]. The κ value is categorized as follows: values between –1 and 0 represent “no agreement”; between 0 and 0.20 represent “poor agreement”; between 0.21 and 0.40 represent “slight agreement”; between 0.41 and 0.60 represent “fair agreement”; between 0.61 and 0.80 represent “good agreement”; between 0.81 and 0.90 represent “very good agreement”; and between 0.91 and 1.0 represent “excellent agreement” [17]. The PPVs are used to estimate the probability of identifying a true incident case among those identified as being new cases [18], calculated using the 4-year LP as the reference time. The overestimation of incident cases decreased with extension of the LP, which reflects the impact of the length of the LP impacts on the number of incident cases. All the CEIs mentioned above were calculated at quarter-year intervals during the first year of the LP and 1-year intervals after the first year, since the number of identified incident cases varied greatly within the first year of the LP in previous studies [7,12,16].

With the retrograde survival function method, survival (ie, being an incident case) means not having a preceding record at the given time. In detail, for each record in this study, let *a* represent the date of the last record of diabetes of a patient in 2020, *b* the date of the most recent previous record in the LP, and *c* the first date of the LP. The retrograde survival time is (*a*–*b*) for patients with preceding records. Patients without preceding records during the LP, indicating survival, are defined as censored cases, with the censored time (*a – c*). Based on the survival time and censored time, we established the survival function. Survival probability, S(t), the probability of surviving at a specific time, is represented by the equation S(t)=P (T>t), where *T* is the year of the LP, calculated using the Kaplan-Meier estimator. Then, we used the following 2 approaches to determine the optimal LP based on the survival function. For the first approach, we constructed a hazard function. The hazard function represents the instantaneous probability of having the end point event during (*t,∆t*) conditional on survival up to *t* or later. In actuarial terms, it is calculated by 
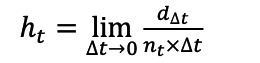
, where *d_∆t_* is the number of patients with the previous record during (*t,∆t*) and *n_t_* is the patient at risk during the interval. Since the retrograde hazard of diabetes will decrease and converge to zero and the survival function will converge to the “true” probability of being incident, we assumed that a time point (*tf*) exists where 
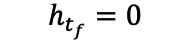
. At this point (*tf*), all cases with preceding records have been found, and the remaining cases are risk-free; this is exactly the optimal LP. To estimate (*tf*), for practical purposes, we defined (*tf*) as the first time point when h_t_ ≤0.0005. For the second approach, we counted the frequency of S(t) corresponding to each day calculated by the retrograde survival function. We assumed that there was a stable S(t), where the frequency was high and increased significantly, representing that S(t) stays at S(*t_f_*) relatively constantly over a long time and that the change in S(t) approaches zero. The t_f_ will be considered as the point when S(t) first reaches S(*t_f_*).

Statistical analyses were performed using SAS (version 9.4; SAS institute).

## Results

After removing records according to the criteria above, 16,132,023 records from medical insurance claims data from 2016 to 2020 were used in this study; these records came from 3,185,337 individuals. There were 3,600,089 records and 247,254 cases of diabetes in total. The insurance record history of 109,476 diabetes prevalent cases in 2020 was screened with the different LPs to identify incident cases. Inclusion and exclusion details are shown in Figure 1, and basic information on the data is shown in Table 1.

**Table 1:** Basic information on the data used in this study (January 1, 2016, to December 31, 2020). “Insurance records” represents all claims and diabetes records in each year. “Individuals with insurance records” represents the number of persons with claims in each year. “Visits per capita” was calculated as the number of insurance records divided by the number of individuals with insurance records.

		Insurance records, n			Individuals with insurance records, n			Visits per capita, n	
		Inpatient	Outpatient	Inpatient		Outpatient	Inpatient		Outpatient
**Entire database**									
	2016	1,227,374	1,326,862	894,726		128,819	1.37		10.30
	2017	1,459,273	1,583,937	1,027,600		151,130	1.42		10.48
	2018	1,484,065	1,815,786	1,024,998		168,547	1.45		10.77
	2019	1,559,320	2,123,446	1,061,351		196,294	1.47		10.82
	2020	1,393,165	2,158,795	947,821		220,560	1.47		9.79
**Diabetes cases**									
	2016	26,597	526,619	23,117		58,703	1.15		8.97
	2017	31,053	618,289	26,833		67,542	1.16		9.15
	2018	31,668	699,739	27,425		75,361	1.15		9.29
	2019	34,131	793,431	29,594		87,197	1.15		9.10
	2020	30,604	807,958	27,038		97,396	1.13		8.30

Applying the longest LP identified 19,086 incident cases of diabetes. Only 17% (19,087/109,476) of the prevalent patients in 2020 were identified as incident cases as they did not have preceding records for diabetes in the longest LP before 2020, revealing that around 80% (90,389/109,476) of patients in 2020 were not truly incident cases. The κ coefficient and PPVs for these results rose with extension of the LP. Agreement was good when a quarter-year LP (κ=0.751) was used, changing to very good (κ=0.899) and excellent (κ=0.950) when the 1-year and 2-year LPs were used. As for PPVs, to achieve a PPV of at least 90% (10% of false positives) [19], an LP of 2 years was necessary. The overestimation rate decreased with the extension of the length of the LP and did so especially sharply during the 1-year LP. After a 2-year LP, the overestimation decreased by under 10%. In general, according to the results of these CEIs, the optimal LP is 2 years for the Chinese population. Detailed information on the CEIs is shown in Table 2.

**Table 2:** Positive predictive values, κ coefficients, and overestimation with different look-back periods, all of which represent the agreement between the incident cases with different look-back periods and the 4-year look-back period as the longest period. Incident cases are defined as those not having previous records in the corresponding look-back period.

LP^a^ (years)	Incident cases, n	PPV^b^, %	κ	Overestimation, %
0.25	29,407	0.674	0.751	0.485
0.5	26,195	0.756	0.825	0.323
0.75	24,172	0.819	0.876	0.220
1	23,288	0.850	0.899	0.176
2	21,472	0.922	0.950	0.084
3	20,468	0.968	0.980	0.033
4	19,806	1	N/A^c^	0

^a^LP: look-back period.

^b^PPV: positive predictive value.

^c^N/A: not applicable.

**Figure 3:**
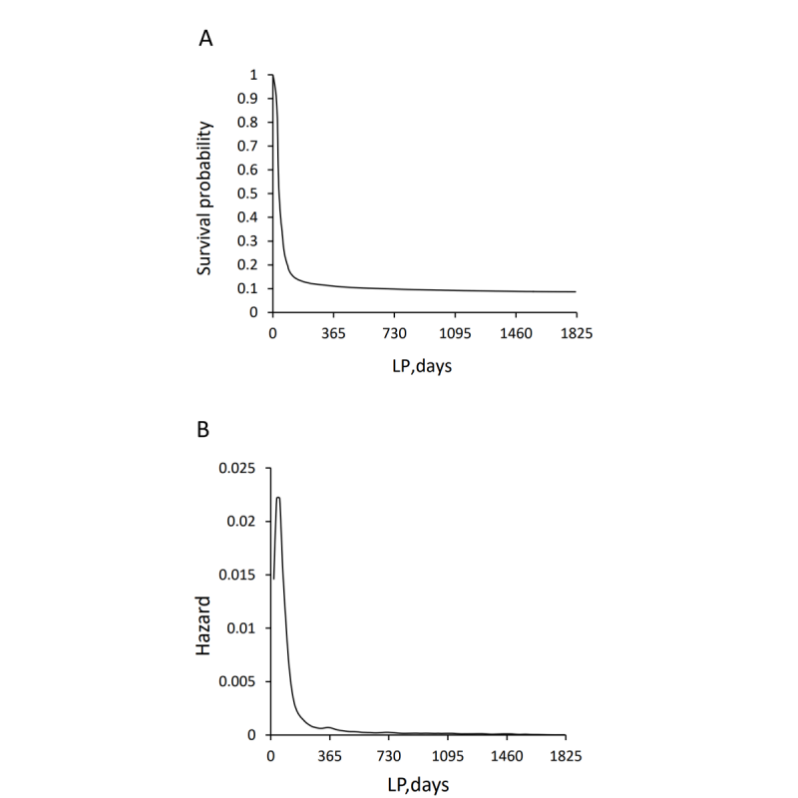
(A) Change in survival probability with LPs with different numbers of days. Survival probability represents the probability of being an incident case at a specific day in the LP, which is calculated using the Kaplan-Meier estimator. (B) Change in survival hazard with LPs with different numbers of days. Survival hazard represents the instantaneous probability of having the end point event. LP: look-back period.

The retrograde survival function graphically represented the exclusion, showing the greatest decrease during the first year of screening (Figure 3). During the first 1-year LP, S(t) dropped from 1.00 to 0.11 (365 days) rapidly. The more days were included in the LP, the more the survival function converged to the “true” probability of being incident [18]. According to the hazard function, the first time point when h_t_ ≤0.0005 was with a 417-day LP: h_t_=0.000459; S(t)=0.11. This meant that the derivative (slope) of the survival function tended toward zero at this time.

**Figure 4:**
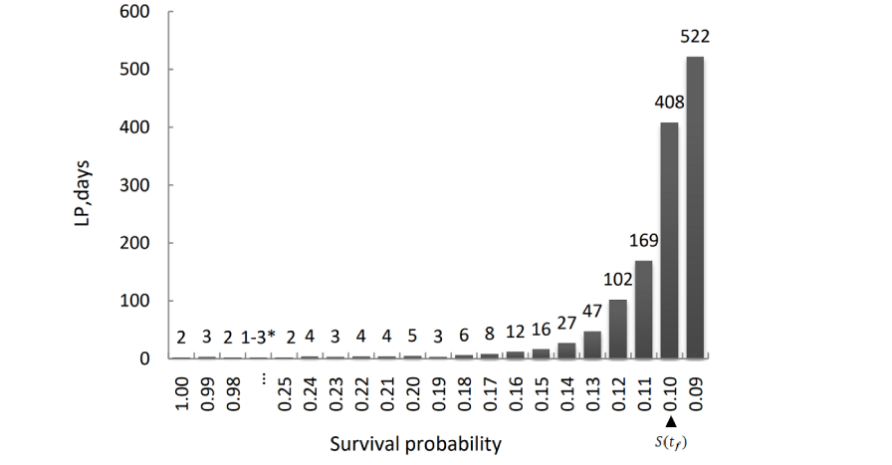
The frequency of S(t). *The frequency of S(t) from 0.97 to 0.26 was between 1 and 3. LP: look-back period.

The results for frequency showed that the frequency of S(t) was small, from 1.00 to 0.13, indicating that S(t) changed greatly. A change in S(t) from 0.11 to 0.10 means that the frequency has significantly increased, from 169 to 408, which is more than double (Figure 4). This means that the risk for a preceding record at this time remained relatively constant at 0.10 over a very long time, and the first day when S(t) reached 0.10 was 480 days. Combining the results for frequency and hazard function, the retrograde survival function method showed that the optimal LP was more than 400 days, which is closest to 2 years.

When using 2 years as the optimal LP, there were 21,472 incident cases. The number of female patients (n=10,862) was a little more than that of male patients (n=10,610), and 84.74% (18,192/21,472) of incident cases were between the ages of 45 and 79 years (Table 3).

**Table 3:** Incident cases applying a 2-year look-back period (ie, using 2018 and 2019 to identify incident cases in 2020) with different ages and genders.

Age group (years)	Male patients, n (n=10,610)	Female patients, n (n=10,862)	Total patients, n (n=21,472)
0-4	4	7	11
5-9	7	16	23
10-14	36	29	65
15-19	34	23	57
20-24	52	45	97
25-29	99	63	162
30-34	308	194	502
35-39	401	178	579
40-44	577	271	848
45-49	961	538	1499
50-54	1432	1073	2505
55-59	1806	1720	3526
60-64	1544	1778	3322
65-69	1504	2061	3565
70-74	1000	1533	2533
75-79	481	764	1245
80-84	269	394	663
≥85	95	175	270

## Discussion

### Principal Results

In our study, a 2-year LP had good CEIs with excellent κ agreement (0.950>0.9), a high PPV (92.2%) and a lower than 10% overestimation rate. As for the retrograde survival function, it showed that a 2-year LP can reliably distinguish new cases in a prevalent pool. According to the results of the methods above, we confirmed that the optimal LP for identifying incident cases of diabetes using the Chinese insurance database provided by the MSB of Weifang is around 2 years, and all the methods are effective and stable.

### Comparison With Prior Work

#### Optimal Length of LP Compared to Other Work

A 2-year LP for diabetes incident case identification using administrative data is the same length as used by a few previous studies [20,21]. A study in Manitoba, Canada, also showed a probability of 0.96 for patients with diabetes to have subsequent medical contact for diabetes within 2 years [22]. However, our results differ from those of Asghari et al [7], who reported an LP of 5 years for health insurance data from Quebec, Canada. A possible reason might be different patterns and medical contact cycles among Chinese and Canadian populations caused by the different medical insurance policies. The hazard function figure (Figure 3B) reflects this difference. In our study, the hazard function was not monotonic, indicating that a few diabetes patients had medical contact irregularly in this database, unlike the Canadian insurance data. n addition, different judgment criteria on CEIs and h_t_ in these studies might have caused the differences.

#### Methodological Comparison

As for retrograde survival function, Brameld et al [15] chose a hazard rate under 0.00001 as the standard when using the retrograde survival method for identifying the optimal LP for diabetes, resulting in a 13-year optimal LP for diabetes among a group of LPs with the longest LP being 15 years, much longer than our study and most other studies [20,23,24]. We think an overly strict standard may have caused an overly long LP. To avoid an overly long estimation of optimal LP, we used h_t_ ≤0.0005 as the criterion, which is similar to the study by Asghari et al [7], which showed that a 2-year LP was optimal for diabetes. That study defined the optimal LP as the time when S(t) was stable and constant [7]; however, the authors did not provide a detailed definition for stable S(t), that is, S(t_f_). In our study, we tried a new approach. We counted the frequency of S(t) and defined S(t_f_) by the frequency of S(t) being greatest and increasing significantly.

Regarding CEIs, such as the κ coefficient, PPVs, and overestimation rate, although they are convenient to calculate and used by many researchers, their criteria are not completely consistent between studies. The overestimation rate has been used by a few researchers to evaluate the impact of varying lengths of LP [11,21,25-27], with values such as 10% [11] and 20% [21]. It is the same for PPVs, as some studies have chosen 80% as the evaluation criterion [19], while some have chosen 90% [28]. The criteria of the κ coefficient have generally between consistent (0.8 or 0.9) [7,18], but some researchers have considered that such high agreement may be related to the high number of prevalent cases of diabetes [7]. Therefore, the effectiveness of κ may remain to be further verified.

Because of the inconsistency of criteria, Beaudet et al [18] chose to combine the results of κ and PPV to identify the optimal LP. In our study, we compared the results of the κ coefficient, PPV, the overestimation rate, and the retrograde survival method. We found that a κ coefficient of 0.9, 90% PPV, 10% overestimation, and 0.0005 hazard rate may be the most suitable criteria, returning stable and constant results, with a 2-year LP as the optimal period.

In general, the CEIs were convenient to calculate, while retrograde survival curves showed the probability of “surviving” converging to the plateau, allowing us to obtain the optimal LP visually and quantitively. Moreover, the criteria of these methods are different. Previous studies have mostly used only one method for identifying the optimal LP, whereas 2 different methods, as used in this study, may lead to more robust results, which could then be referred to when analyzing LPs for other diseases in the administrative data.

### Number of Incident Diabetes Cases

In our study, there were 109,467 diabetes cases in 2020 before LP adjustment. After using the 2-year LP, the number of incident cases decreased to 21,472, which means this adjustment is important. In addition, our study showed that 84.74% (18,192/21,472) of incident cases were between the ages of 45 to 79 years, indicating that the middle-aged and older population has a high incidence of diabetes. We should make efforts to focus on this population to prevent diabetes.

### Innovativeness

Our study is the first to identify 2 years as the optimal LP for diabetes in the Chinese population when using insurance data, which supports efforts to estimate the incidence of diabetes among populations using insurance data. More importantly, we used 2 different methods to identify the optimal LP, a method that could be referred to when analyzing LPs for other diseases in administrative data.

### Limitations

Our study has some limitations. In our database, without more detailed records for disease classification, it was not possible to differentiate between type 1 and type 2 diabetes. However, there are far fewer patients with type 1 diabetes than with type 2 diabetes in the overall population. Thus, we considered the study population to be generally homogeneous and that these results may be representative and stable. Furthermore, we only applied up to a 4-year LP. Although the results of the retrograde survival function analysis showed stabilization of risk of being a preceding case after a 2-year LP, we expect to use longer LPs in future studies with more accumulated data.

### Conclusion

All CEIs (the κ coefficient, PPV, and overestimation rate) and the retrograde survival function method can effectively identify the optimal LP for diabetes incident cases, with stable and constant results. We found that a 2-year LP was optimal to remove preceding diabetes cases and identify the true incidence of diabetes using medical insurance data in the Chinese population.

## Abbreviations

CEI: consistency examination index
ICD-10: International Classification of Diseases, 10th Revision
LP: look-back period
MSB: Medical Security Bureau
PPV: positive predictive value

## Data Availability

The insurance database used in this study is not publicly available due to a confidentiality agreement with Medical Security Bureau of Weifang but it is available from the corresponding author on reasonable request.
